# MicroRNAs Regulate Intestinal Immunity and Gut Microbiota for Gastrointestinal Health: A Comprehensive Review

**DOI:** 10.3390/genes11091075

**Published:** 2020-09-12

**Authors:** Kefan Bi, Xujun Zhang, Wenbiao Chen, Hongyan Diao

**Affiliations:** State Key Laboratory for Diagnosis and Treatment of Infectious Diseases, National Clinical Research Center for Infectious Diseases, Collaborative Innovation Center for Diagnosis and Treatment of Infectious Diseases, The First Affiliated Hospital, Zhejiang University School of Medicine, Hangzhou 310003, China; bdgxbkf@zju.edu.cn (K.B.); 290516@zju.edu.cn (X.Z.); 11718164@zju.edu.cn (W.C.)

**Keywords:** microRNA, intestinal immunity, gut microbiome, gastrointestinal disease

## Abstract

MicroRNAs are small non-coding RNAs regulating gene expression at the post-transcriptional level. The regulation of microRNA expression in the gut intestine is gradually recognized as one of the crucial contributors of intestinal homeostasis and overall health. Recent studies indicated that both the microRNAs endogenous in the gut intestine and exogenous from diets could play influential roles in modulating microbial colonization and intestinal immunity. In this review, we discuss the biological functions of microRNAs in regulating intestinal homeostasis by modulating intestinal immune responses and gut microbiota. We particularly focus on addressing the microRNA-dependent communication and interactions among microRNA, gut microbiota, and intestinal immune system. Besides, we also summarize the roles of diet-derived microRNAs in host-microbiome homeostasis and their benefits on intestinal health. A better understanding of the relationships among intestinal disorders, microRNAs, and other factors influencing intestinal health can facilitate the application of microRNA-based therapeutics for gastrointestinal diseases.

## 1. Introduction

MicroRNAs are a class of small non-coding RNAs, 20–22 nucleotides in length, located at intragenic and intergenic regions of the genome, and they establish a diverse and complex regulatory network in regulating gene expressions and participating in various biological functions [1]. MicroRNAs are transcribed by RNA polymerase II as long primary transcripts called primary miRNAs (pri-miRNAs), which contain hairpin and flanking sequences. The hairpin structure of pri-miRNA is subsequently cleaved by RNA polymerase III. Drosha and essential cofactor DGCR8 liberate one or more 70–90 nucleotide hairpin in the pre-miRNA, which are transported to the cytoplasm for further cleavage by Dicer enzymes to form a mature miRNA. The mature miRNA is subsequently incorporated into the miRNA induced silencing complex (miRISC), and can bind to the specific UTR of mRNA molecules with complementary sites, which ensures that the miRNA can silence gene expression. It is suggested that microRNAs can silence a wide range of mRNA expression though translational repression or stimulation of the degradation of the mRNA. miRISC induces mRNA degradation and translational repression by attaching to the 3′ untranslated region of the target mRNAs in an incomplete or a complete complementary manner [2,3,4,5]. MicroRNA play a powerful and essential role in post-transcriptional gene regulation, which contributes to cell differentiation, proliferation, and apoptosis, as well as being involved in the development of multiple gastrointestinal diseases [6].

The human gut intestine is colonized by complex symbiotic microflora, carrying approximately 10^14^ microbes, and these microbes reside in the digestive tract and play critical roles in digestion-absorption functions, as well as a wide range of other physiological processes [7]. These microbes are usually dynamic and sensitive to multiple host factors, including age, genetics, diet, antibiotic treatment, intestinal infection, and immune system. Alterations of the intestinal microbiota are thought to be an important factor in intestinal diseases or extra-intestinal disease1 [8]. Recently, a growing number of research studies have been carried out on investigating the mechanisms by which intestinal microbiota influence human diseases, and related studies have shown that microRNA could play an important regulatory role in human-microbiota interactions [9,10,11].

Intestinal epithelium builds the bridge among intestinal microbes, facilitating their symbiosis with the intestinal immune system for their functions on antigen presentation, type 2 immunity stimulation, differentiation and maturation of immune cells in lamina propria and mucosa-associated lymphoid tissues, and triggering downstream immune responses [12].

A series of studies have revealed that intestinal microbiota and microRNAs may interact with each other for regulating host gene expression [10,13]. In fact, microRNAs have a wide range of effects on the intestinal immune system, and also play important roles in the pathogenesis of bowel diseases [14]. Recently, host microRNAs have also been suggested to participate in the invasion and infection of intestinal pathogens in the gastrointestinal tract [15]. Furthermore, a growing number of studies have demonstrated the functional roles of microRNAs in connecting the communication between intestinal microbes and the host intestinal epithelial cells [16,17].

It has been identified that most fecal microRNAs are derived from gut epithelial cells, especially Hopx-expressing cells, which can influence the composition and distribution of intestinal microbiota [11]. For instance, miR-101, miR-515-5p, miR-876-5p, miR-325, miR-1253, miR-1224-5p, miR-1226-5p, and miR-623 can all modulate and promote the growth of specific bacteria in the gut intestine [11]. In addition, recent studies suggested that fecal microRNAs are potential biomarkers for intestinal diseases, such as colorectal cancer and inflammatory bowel disease (IBD) [18,19]. The triangle relationships among microRNAs, gut intestinal microbiota, and host immunity are decisive for the homeostasis or dysbiosis of the gastrointestinal environment [20].

**Figure 1:**
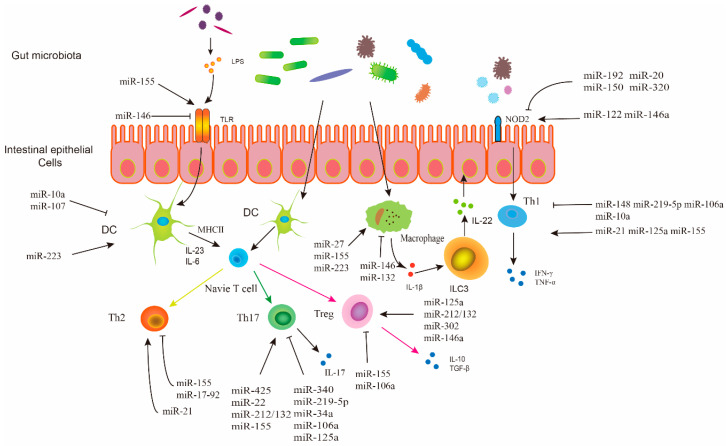
Effect of microRNA on the regulation of immune cells in the intestine.

In the following sections of the review, we summarize and discuss the studies about the bio-functions of microRNAs in the gut intestinal microenvironment, in which they serve as the link between the intestinal immune system and microbial community. The representative microRNAs active in influencing intestinal immunity and homeostasis are summarized in Table 1 and Figure 1, and their regulatory roles in the gastrointestinal tract are further addressed. Moreover, the modulatory effects of exogenous microRNAs on the gut intestinal microbiome are also described. Additionally, the potential of microRNAs in the gut intestine as biomarkers and candidate therapeutic targets for gastrointestinal diseases is briefly discussed as well.

## 2. MicroRNA in Regulating the Intestinal Immune System

### 2.1. MicroRNA and Innate Intestinal Immunity

The homeostasis of the intestinal environment depends on several factors, including host genetics, intestinal immune system, gut microbiota and metabolites, and intestinal barrier integrity and functions [30]. In terms of the host defense against intestinal pathogens, the mucosa-associated immune system protects the gut intestine at an early stage, through the dynamic functions and coordinated cellular interplays of epithelil cells, dendritic cells, and macrophages for recognizing foreign invaders [31,32]. Moreover, intestinal commensal bacteria are essential for the maturation of innate immune system. In fact, the interactions between dendritic cells and natural killer cells can initiate the intestinal immune responses by activating dendritic cells and macrophages via different pathogen-associated molecular patterns [33,34]. A number of recent studies have revealed that microRNA has a significant impact on immune cells and immune responses, defense against pathogenic microorganisms [30], intestinal mucosal barrier, and the development of intestinal epithelial cells [35], which largely influence intestinal homeostasis, and are extensively associated with autoimmune diseases. The innate immune system is considered as the first line of defense against foreign antigens and pathogens [36]. Growing evidence suggests that microRNA is indispensable for determining the fate of epithelial cells, such as proliferation, differentiation, or autophagy, through influencing targeted signaling pathways [35,37,38]. For example, the Wnt signaling pathway is closely associated with the mucosal immune homeostasis and intestinal epithelial differentiation [39]. Recently, miR156 was identified to possess essential functions in inhibiting intestinal cell proliferation through regulating Wnt/β-catenin signaling pathway, in which miR156 could down-regulate the expression of Wnt10b and thus up-regulate β-catenin phosphorylation in the mouse model [24]. In another study, miR-31 was found to enhance the regeneration of epithelial cells through regulating the Wnt/Hippo signaling pathway, suppressing immune responses, and thus promoting the proliferation of intestinal epithelial cells for counteracting intestinal inflammatory diseases [23]. Moreover, the inflammatory-induced intestinal secretion of miR-31 could suppress the expression of several receptors such as GP130, IL17RA, and IL7R, which are involved in the activation of STAT3, and prevent the disruption or injury of intestinal epithelium [23]. The overexpression of miR-146a in the intestinal epithelial cells to response to the bacterial antigenslike lipopolysaccharide (LPS) or cytokines depends on the activation of the TLR4/MyD88/NF-kB, can induces immune tolerance and inhibits cytokine production in response to LPS and IL-1β in intestinal epithelial cells in the mouse model with colitis. [40]. Moreover, miR-375-3p is one of the microbiota-sensitive microRNAs that can regulate the proliferation of intestinal epithelial stem cells (IESCs). It has been found that miR-375-3p is highly expressed in IESCs and can act as a regulator for reducing IESC proliferation [41].

The defensive system of innate intestinal immunity against invading gut pathogens is initiated by pathogen-associated molecular patterns (PAMPs); microRNAs are significantly connected with two general classes of PAMPs, nucleotide-binding oligomerization domain-containing protein (NOD) 2 and Toll-like receptor (TLR) [42,43].

NOD2 is a member of the NOD-like receptor family that encodes on human chromosome 16 and can recognize muramyl dipeptide, which functions as an intracellular sensor for pathogens [44]. NOD2 is considered as one of the genetic risk factors in the pathogenesis of IBD, and it is also recognized as the strongest single genetic susceptibility locus in Crohn’s disease [45]. The biogenesis of microRNAs and their functional roles in the adjustment and regulation of NOD2 are regarded as the critical factors for the initiation of innate immune responses [44]. The interaction between NOD2 and microRNAs, such as miR-20, miR-122, miR-192, miR-146a, and miR-320, is generally recognized to be prevalently connected with the pathogenesis of IBD [38,46,47,48,49]. MiRNAs regulate intestinal homeostasis by interacting with NOD2, targeting on intestinal epithelial cells and activating immune cells [44,50]. Pierdomenico et al. indicated that miR-320 can target NOD2, and that its expression was negatively correlated with NOD2 expression in IBD patients [51]. Further, the downregulation of miR-320 can also upregulate NF-κB activity and inflammatory cytokine production in the HT-29 cell lines. On the contrary, NOD2 can also regulate the expression of miR-21 in dendritic cells for the secretion of IL-23, the immunomodulatory mechanism of which is involved in the pathogenesis of Crohn’s disease [50].

TLR works as the sensor of innate immunity by identifying PAMPs and detecting invaded pathogens, which is highly related with the abnormal immune responses in the patients of IBD [52]. MicroRNAs are considered as the collaborators and regulators of the activation of TLRs. For instance, the up-regulation of miR-146a-5p expression was induced upon TLR4 stimulation by LPS for activating the downstream NF-κB signaling pathway, which plays an essential role in host intestinal defense against microbial infections [53]. Zhen et al. reported that let-7b/TLR4 signaling pathway could be activated by the infection of adherent invasive *Escherichia coli*. The down-regulation of let-7b resulted in the promotion of TLR4 and increased the secretion of pro-inflammatory factors, such as IL-6, IL-8, and TNF-α, for further stimulating intestinal epithelial cells and aggravating intestinal inflammation [54].

### 2.2. MicroRNA and Adaptive Intestinal Immunity

The maturation of the intestinal adaptive immune system is controlled by a complex network of signaling, and the differentiation and formation of nearly all the adaptive immune cells, for example, T cells and B cells, can be regulated by host microRNAs in the gut intestine [55]. The aberrant expression of these microRNAs leads to immunological abnormality and even autoimmune diseases [56]. Naive T cells within the intestines and lymph node can differentiate into different subtypes of T cells. Intestinal microbiota play important role in various processes of T cell development, both within and outside the intestines. Several specific bacterial species can induce the differentiation of T cells into particular subtypes [56,57]. Th17 and Treg cells are particularly important to regulate autoimmunity in the intestines. Th17 cells are significantly correlated with both ulcerative colitis and Crohn’s disease, and they induce the expression of transcription factor RORγt+ for producing cytokines associated with IBD, such as IL-17, IL-22, and IL-23 [58,59,60,61]. Regulatory T (Treg) cells are a group of CD4+ T cells that could regulate the activity of T cells and the entire immune system in gut intestine, and play an important role in the inhibition of the secretion of inflammatory cytokines and suppressing excessive immune responses.

CD4+ T cells can recognize and adapt to antigens or pathogens to be differentiated into different T cell subsets. T-bet, found in Th1 cells, is the key regulator of Th1 development and function, which enables them to produce type 1 cytokines, including IFN-γ, IL-6, and IL-12 [62]. Continuous GATA3 expression is essential for CD4+ T cells to differentiate into Th2 cells, and induces the GATA3-mediated Th2 cytokine production (IL-4, IL-5, and IL-13) [63].

According to previous studies, Crohn’s disease is highly related to Th1, while ulcerative colitis tends to be driven by Th2 [64,65]. Intestinal microRNAs can actually participate in these two intestinal diseases by regulating the differentiation of CD4^+^ T cells. Wu et al. indicate miR-17-92 can regulate the differentiation of the CD4+T cell to Th1 rather than Th2 though the overexpression of miR-17-92 can increase the production of IFN-γ to promote the CD4+T cell differentiation. Meanwhile, the over-expression of miR-17-92 can stimulate the secretion of IFN-γ, which further promotes CD4^+^ T-Th1 cell differentiation. Intestinal miR-146a, miR-29, miR-128, and miR-126 have been demonstrated to influence the induction of Th1 in gut intestine [66,67,68,69]. Moreover, the over-expression of intestinal miR-155 can promote CD4^+^ T-Th1 cell differentiation, the knockdown of miR-155 can inhibit CD4^+^ T cells from distributing to the effector sites in human colon, and miR-155 knockout can facilitate CD4^+^ T-Th2 cell differentiation [70,71,72]. Additionally, intestinal miR-21 has been observed to induce CD4^+^ T-Th2 cell differentiation, thus participating in the pathogenicity of IBD [46].

The differentiation of Th17 is an important regulator for intestinal homeostasis, which can be manipulated by responding to resident microflora or the microRNAs through different regulatory mechanisms [72]. For instance, intestinal miR-155 has been identified to promote cytokine secretion by dendritic cells for further regulating Th17 cell differentiation and immunologic functions, which were closely associated with the development of chronic intestinal inflammation and autoimmune diseases [73]. On the contrary, miR-155 knockdown reduces Th17 cell density and induces functional defects of inflammatory cytokine production, which further targets aryl hydrocarbon receptors for suppressing mucosal immune responses and ameliorates colitis [74]. Furthermore, miR-106a, miR-18b, and miR-363-3p can inhibit Th17 differentiation and reduce the secretion of cytokines, especially IL-17, in the gut intestine [75]. MiR-125a is highly expressed in intestinal T cells for regulating their functions, and a down-regulated miR-125a expression can influence Th1/Th17 cell differentiation in intestinal immune responses in mouse model [76]. This effect is reflected by the mice with miR-125 knockout, for which the induced Th1 and Th17 cell infiltrations severely damage the intestine barrier and stimulate colitis [76]. Moreover, miR-34a can protect the intestine from *Citrobacter*-induced colon cancer through targeting IL-6R and IL-23R for inhibiting the differentiation of Th17 cells and their infiltration, which suppresses the inflammation-induced proliferation of cancer stem cells [77].

It has been revealed that intestinal microRNAs also act as influencers for the differentiation and maturation of T_reg_ cells. Based on the mice model, the antibiotic-induced alteration of intestinal microbiome influences the secretion of intestinal microRNAs, which may, in turn, affect immune-regulatory armament and energy metabolism in the gastrointestinal tract [78]. MiR-141 or miR-200a are demonstrated to be able to regulate Th17 cell differentiation and T_reg_ cell formation in MS patients, thus suppressing autoimmune diseases in the intestines [79]. MiR-155 can regulate Th17/T_reg_ cell balance by inhibiting cytotoxic T-lymphocyte-associated protein 4 expressions, and the overexpression of miR-155 reduces follicular T_reg_ cells, while central T_reg_ cells and miR-155-5p further target sirtuin-1 to maintain the balance of Th17/T_reg_ cells [80]. Furthermore, miR-122a, miR-146a, miR-155, and miR-181a are highly expressed in T cells, with a close connection with T_reg_ cells, which can target interferon regulatory factor 4 for suppressing IL-6 secretion and inducing IL-17A production, thus assisting in the mitigation of IBD [81,82,83,84]. In addition, the overexpression of miR-125a can inhibit the differentiation of CD4^+^ T cells into either Th1 or Th17 cells, which also contributes to immune-regulation [85].

## 3. MicroRNA in Modulating Gut Intestinal Microbiota

### 3.1. MicroRNA-Microbiota Interaction in Gut Intestinal Health and Disease

**Figure 2:**
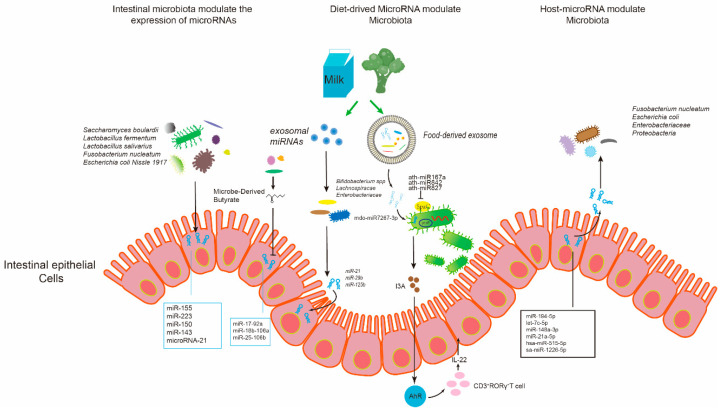
Summary of microRNA-microbiota interaction in gut intestine, and the crucial role in regulating intestinal homeostasis through multiple mechanisms. The microRNAs secreted by intestinal epithelial cells are released into lumen, modulating the growth of gut microbes and the composition of intestinal microbiota. Gut microbiome in the host can be influenced by diet-derived microRNAs as well, while gut intestinal microbiota may in turn regulate the expression of intestinal microRNAs in the host.

The intestinal immune system, gut microbiota, and mucosal barrier are all critical components in maintaining the dynamic balance of intestinal microenvironment, while gut microbial communities significantly contribute to the complex and tightly regulated system in dynamic intestinal equilibrium [86,87,88]. Probiotics are crucial parts of gut microflora, which can inhibit and exclude the colonization of pathogenic bacteria through various mechanisms [89,90,91]. For example, they possess unique roles in maintaining and activating intestinal immunity, especially mucosal immune responses [92]. Through activating innate or adaptive immunity in the gastrointestinal tract, probiotics can competitively inhibit pathogens attacking intestinal mucosa and enhance the integrity of the intestinal epithelial barrier [92,93]. They interact with the intestinal epithelial cells for promoting mucosal immunity by initiating pro-inflammatory or anti-inflammatory responses, which overall contributes to maintaining the homeostasis of gut intestine [86]. Recently, there have been several progresses in identifying the roles of endogenous intestinal microRNAs in manipulating gut microbiota, especially pathogenic bacteria and commensal flora, which are associated with the progression of intestinal diseases [94]. In Figure 2 and Table 2 we summarize the association between intestinal microbiota and the miRNA expression in the host intestine, and the prominent role in regulating intestinal homeostasis.

Previously, Guillaume et al. uncovered the potential mechanism under the intestinal microbiota regulation on host gene expression, which is possible via the connector microRNAs [11]. In another study, the differentially expressed microRNAs in germ-free and conventionally raised mice are connected with intestinal cell communication, signal transduction, inflammatory responses, and the influences on mucosal barrier-related gene expression, which further supports that the endogenous gut microbiota associates with microRNAs in modulating intestinal gene expression [95]. The alteration in the structure and abundance of gut microbiota will result in an altered intestinal miRNA profile, evidence indicates that gut microbiome can impact colon epithelial cells in several ways, like DNA damage, DNA methylation, change the chromatin structure, especially the relationship between the gut microbiome and non-coding RNAs [96].

Furthermore, microRNAs mediate the modulation of gut microbiome composition and their secretion of secondary metabolites; the aberrant microRNA expression and dysbiosis of gut intestinal microbiota are strongly correlated with a variety of intestinal diseases, particularly IBD and colorectal cancer [97,98,99]. Studies on miR-194-5p, miR-148-3p, and miR-27b-3p reveal that these microRNAs could be intermediates between Enterobacteriaceae and gut inflammation, and they have a strong correlation with Proteobacteria, which were previously reported to be abundant in IL-10 knockout mice and IBD patients, indicating their roles as mediators in the pathogenesis of Proteobacteria and intestinal inflammation [11,100]. Relying on fecal microbiota transplantation, the germ-free mice received intestinal microbes from wild-type donor, which exhibited a significant difference in their fecal microRNA profiles, and several other microRNAs, such as miR-144, miR-519, and miR-211, have been considered as important modulators of gut microbiota and biomarkers for Crohn’s disease, verified in the adult CD patients [101]. Moreover, in a study based on the DSS-induced colitis mice model, miR-21 was mentioned as a factor for the pathogenicity of intestinal inflammatory diseases and colitis-associated colorectal cancer, primarily through stimulating IL-10 and prostaglandin E2, which further inhibit anti-tumor adaptive immunity [102]. While *Fusobacterium nucleatum* can significantly up-regulate the expression of miR-21 through TLR4/MYD88/NF-κB pathway and facilitate the progression of colitis-associated colorectal cancer [102], in CRC patients on the other side, intestinal microbiota can also influence the host gene expression of intestinal microRNAs, which contributes to protecting intestinal homeostasis and attenuating gut inflammation [103,104]. For example, two probiotic strains, *Lactobacillus fermentum* and *Lactobacillus salivarius*, can restore the down-regulated expressions of miR-155 and miR-223, which ameliorate microbiota dysbiosis, preserve the function of the mucosal barrier, and relieve the DSS-induced colitis in mice model [105].

### 3.2. Diet-Derived MicroRNAs as Modulators for Gut Microbiome

Diet has been suggested to serve as the main modulator of host’s gut microbiota, since food is the major energy source, for not only the host, but also the intestinal microbes [113,114,115]. A large number of experimental evidence and clinical outcomes have indicated that diet is also one of the most important regulators, either directly or indirectly, for host microRNA expression in the intestine [116], which includes the microRNAs derived from dietary sources or food metabolites [117]. Interactive analysis of the relevance between intestinal microbiome and microRNAs obtained from diet revealed that miR-21, miR-146a, and miR-155 are potential factors coexistent in the host and foods, which play central roles in cellular senescence, DNA damage, and inflammatory reactions [118,119]. It is also suggested that dietary fibers in cruciferous vegetables could be utilized by intestinal microbes for the generation of trace elements and secondary metabolites, which further change the host intestinal microRNA profiles, regulate cancer-related microRNA expression, and eventually inhibit multiple cancer-related oncogenic signaling pathways [120,121]. Moreover, the microRNA profiles in peripheral blood could serve as biomarkers for evaluating individual nutritious status and diet habits [122]. Studies have also found that water intake can improve epithelial cells and mucosal barriers in the gastrointestinal tract by up-regulating the content of miR-1968-5p.MiR-1968-5p can target the MyD88 to inhibit the expression of the MyD88 in intestinal epithelial cells for reducing intestinal inflammation, which modulates the community of host intestinal microflora in mouse model [123].

Extracellular vesicles are present in most types of eukaryotic and prokaryotic cells, which contribute to carrying and transporting proteins, RNAs (mRNA, microRNA, lncRNA, and other RNA species), and DNAs [124]. They facilitate the absorption of microRNAs from the digested external diet in the digestive tract of the host under various conditions [125]. The exosomes protect the integrity and stability of microRNAs from detrimental intestinal environments, such as digestive enzymes and low pH, by surrounding them with extracellular phospholipid bilayer vesicles [125]. Moreover, exosomes carrying microRNAs from milk are absorbed by intestinal epithelial crypt-like cells through the digestive system. The microRNA rich in the breast milk has been confirmed closely related with inflammation and immune response [126], which benefit the intestinal immunity and nutrients of infants, as well as the growth of preterm infants during early development [127].

It was indicated that plant-derived exosomal microRNAs could influence intestinal homeostasis and serve as potential therapeutic targets for IBD and colorectal cancer [128]. A recent study has shown that exosomal microRNAs generated after intestinal absorption of a plant-sourced diet can regulate and shape the composition and distribution of gut microbiota, which further suppresses gut inflammation and restore intestinal barrier for colitis inhibition in mice [112]. Furthermore, diet-derived microRNAs can crosstalk with gut intestinal microbiome for maintaining the overall healthy gastrointestinal environment. Analysis on the expression profiles of intestinal microRNAs indicates that diet with resistant starch can induce the down-regulation of colonic oncogenic miR-17-92, which significantly enriches *Ruminococcus Bromii*, *Lactobacillus gasseri*, and *Parabecteroides distasonis*, while it reduces the relative abundance of *Bilophila* and *Sutterella*, promoting the overall butyrate production by gut microbiota, and limiting the negative effects brought on by a high-meat diet in Rat model [129,130].

### 3.3. MicroRNAs Mediate the Crosstalk among Intestinal Epithelial Cells, Gut Microbes, and Intestinal Immune Responses

Previous studies have shown that microRNAs are considered as significant regulators in the maintenance of epithelial barrier function [131], cell apoptosis [132], cell proliferation and differentiation [133,134], and immune functions [135,136]. It was found that microRNAs can trigger apoptosis and differentiation of intestinal epithelial cells and immune cells, which modulate the overall intestinal immunity for maintaining a delicately balanced status of intestinal environment [137,138,139]. In addition, it has also been reported that the microRNAs secreted by host intestinal cells can regulate the growth of microbes and the abundance of intestinal microflora via DNA exchanging, impact the target bacteria at the DNA level. Liu et al. have found that microRNA secreted from intestinal epithelial cells can bind to single-stranded DNA target nucleotide sequences in the bacteria, to regulate the bacterial gene transcripts, bacterial growth and motility [11]. Meanwhile, gut microbiota can reversely modulate the host intestinal microRNAs and regulate intestinal epithelial tight junction proteins, which ultimately function in regulating innate and adaptive intestinal immunities, in the in mouse models with colitis and IEC cell lineages [140]. MiR-146a is expressed in ileum and distal colon, which has been demonstrated to be the key molecule for the crosstalk between intestinal immune responses and gut microbiota by restricting the expansion of Th17 and T_reg_ cells and modulating intestinal microflora [40]. Moreover, miR-146a in intestinal epithelial cells can be potently induced by pro-inflammatory cytokines and LPS through TLR4/MyD88/NF-kB-Akt signaling, for inhibiting the activation of intestinal immunity and facilitating the immune tolerance of intestinal epithelial cells [40].

Intestinal microbiome can efficiently communicate with immune system and regulate host microRNA expression to modulate intestinal homeostasis [141]. Previous research has shown that intestinal microbiota can negatively regulate the expression of miR-10a in dendritic cells through interacting with the TLR signaling pathway [142], validated in IBD patients and human primary DC cells, eventually maintaining intestinal homeostasis and suppressing the development of chronic IBD by targeting IL-12/23p40 expression [143]. Johnston et al. previously reported that miR-21-deficient mice could reduce intestinal inflammation during acute colitis, which is attributed to the alterations of intestinal microflora composition [144]. In addition, the oral administration of miR-30d can target and regulate β-galactosidase AMUC_RS06985 expression in *Akkermansia muciniphila* for promoting the expansion of *Akkermansia* abundance in gut, which further stimulates T_reg_ cells to suppress experimental autoimmune encephalomyelitis, indicating the potential of miR-30d for the treatment of multiple sclerosis [145]. Nutrients in milk have been proved to play a role in infant development by regulated gene expression. Milk rich in lactoferrin plays essential roles in infant development, cell proliferation and shape immune functions. Some studies have shown that lactoferrin can regulate the crypt cell transcriptome and is highly related to the secretion of Osteopontin, critically involved in NKT cell function, plays a role in the immune response [127,146]. In addition, intestinal microRNAs are also involved in gut intestinal microbial infections and subsequent immune responses. For example, *Salmonella* can modulate the intestinal miR-128 expression to reduce macrophage recruitment through regulating M-CSF, thus avoiding intestinal immunity for survival and infection [147]. *Listeria* can also inhibit the expression of several intestinal microRNAs, such as miR-192, miR-200b, and miR-215, while *Lactobacillus paracasei* CNCM I-3689 and *Lactobacillus casei* BL23 are able to rescue these microRNA levels during *Listeria* infection, for maintaining the effective intestinal immunity and overall intestinal homeostasis [148]. Besides, the up-regulation of miR-146 in macrophages can also be induced by *Listeria monocytogenes* infection, which then mediates intestinal innate immune responses, signal transduction, and autophagy [149].

## 4. Conclusions

Intestinal microRNAs have been suggested as key players in the maintenance of healthy gastrointestinal environment. On the one hand, these microRNAs regulate the intestinal immune system. For example, they influence innate intestinal immunity through regulating NOD2 and TLR, two of the crucial PAMPs; different microRNAs can also facilitate the differentiation of Th1, Th2, Th17, or Treg cells, thus participating in adaptive intestinal immunity. Intestinal microRNAs from intestinal epithelial cells or external diets interact with gut microbes, and modulate the composition and distribution of the intestinal microbial ecosystem. The complex but efficient crosstalk among gut microbiota, intestinal epithelial cells, and intestinal immunity, mediated by microRNAs, is critical for maintaining the overall gut intestinal health and preventing/ameliorating gastrointestinal diseases such as IBD and colorectal cancer (Figure 1). Therefore, the targeted intervention of microRNAs might be potential therapeutics for dysbiotic intestinal microbiome and chronic gut inflammation.

## Figures and Tables

**Table 1:** Representative microRNAs and their functions in gut intestine.

MicroRNA	Target	Function	Reference
miR-21	Rho-associated protein kinase 1	Regulates tight junction proteins; Protects intestinal barrier from dysfunction	[21]
miR-21	PTEN/PI3K/Akt signaling pathway	Regulates intestinal tight junction permeability; Preserves intestinal barrier	[22]
miR-31	Wnt/Hippo signaling pathway; GP130; IL7R; IL16R	Promotes intestinal epithelial cell proliferation	[23]
miR156	Wnt/β-catenin signaling pathway	Inhibits intestinal cell proliferation	[24]
miR-181c	TNF-α	Regulates TNF-α; Reduces intestinal barrier injury	[25]
miR-191a	Zonula occludens-1	Regulates tight junction proteins; Reduces TNF-α-induced injury	[26]
miR-200b	Myosin light chain kinase	Inhibits TNF-α-induced IL-8 secretion; Suppresses tight junction disruption	[27]
miR-212	Zonula occludens-1	Regulates tight junction proteins; Reduces gut leakiness	[28]
miR-301a	BTG anti-proliferation factor 1	Stimulates NF-κB activation; Promotes mucosal inflammation and tumorigenesis	[29]

**Table 2:** MicroRNA-microbiota interaction in the host intestines.

Bacteria/miRNA	Target	Effect	Reference
*E. coli* Nissle 1917	miR-223, miR-155 miR-150 miR-143, miR-375	Up-regulation Down-regulation	[106]
*Saccharomyces boulardii*	miR-155, miR-223	Down-regulation	[107]
*L. fermentum*	miR-150, miR-155, miR-223 miR-143	Down-regulation Up-regulation	[105]
*L. salivarius*	miR-155, miR-223	Down-regulation	[105]
*Listeria monocytogenes*	miR-143, miR-148a miR-200b, miR-200c	Down-regulation	[108]
*F. nucleatum*	microRNA-21	Up-regulation	[109]
*L. acidophilus* *B. bifidum*	miR-135b, miR-155 miR-26b, miR-18a	Down-regulation Up-regulation	[110]
*B. longum*	miR-145, miR-15a miR-146a	Up-regulation Down-regulation	[111]
miR-515-5p	*F. nucleatum*	Promoting bacterial growth	[11]
miR-1226-5p	*E. coli*	Promoting bacterial growth	[11]
gma-miR396e	*L. rhamnosus*	Promoting bacterial growth	[112]
